# Synthesis and Evaluation of the Cytotoxicity of a Series of 1,3,4-Thiadiazole Based Compounds as Anticancer Agents

**Published:** 2013-11

**Authors:** Alireza Aliabadi, Elham Eghbalian, Amir Kiani

**Affiliations:** 1 Department of Medicinal Chemistry, Faculty of Pharmacy, Kermanshah University of Medical Sciences, Kermanshah, Iran; 2 Students Research Committee, Kermanshah University of Medical Sciences, Kermanshah, Iran; 3 Department of Pharmacology, Toxicology and Medical Services, Faculty of Pharmacy, Kermanshah University of Medical Sciences, Kermanshah, Iran

**Keywords:** Anticancer, Cytotoxicity, Synthesis, 1, 3, 4-Thiadiazole

## Abstract

***Objective(s):*** Nowadays, cancer is an important public health problem in all countries. Limitations of current chemotherapy for neoplastic diseases such as severe adverse reactions and tumor resistance to the chemotherapeutic drugs have been led to a temptation for focusing on the discovery and development of new compounds with potential anticancer activity.

***Materials and Methods:*** A new series of 1,3,4-thiadiazole-derived compounds (3a-3l) were synthesized. *N*-(5-Mercapto-1,3,4-thiadiazol-2-yl)-2-(4-methoxyphenyl) acetamide (2) was prepared through direct amidation of 4-methoxyphenylacetic acid (2) with 5-amino-1,3,4-thiadiazole-2-thiol using EDC (*N*-Ethyl-*N*-dimethylaminopropyl carbodiimide) and HOBt (Hydroxybenzotriazole). Then, various derivatives of benzyl chloride containing electron withdrawing and electron donating moieties were reacted with compound 2 to prepare compounds 3a-3l. *In vitro* cytotoxicity assessment using MTT method was applied and results are presented as IC_50_.

***Results:*** All the synthesized compounds were characterized by ^1^H-NMR and IR spectroscopy. Some of the synthesized compounds were also characterized using MS spectroscopy. Related melting points were also recorded. According to the obtained data from MTT assay, all compounds (3a-3l) demonstrated a higher cytotoxic activity against MDA-MB-231 breast cancer cell line in comparison with other cell lines.

***Conclusion:*** It is notable that four synthesized compounds 3h (IC_50_= 11 ± 0.18 µM), 3j (IC_50_= 10 ± 0.39 µM), 3k (IC_50_= 11 ± 0.77 µM) and 3l (IC_50_= 8 ± 0.69 µM) exhibited higher cytotoxic activity against MDA-MB-231 cell line compared to imatinib (IC_50_= 20 ± 0.69 µM) as the reference drug.

## Introduction

Cancer is a general name for a group of more than 100 diseases in which cells belonging to a part of the body, begin uncontrolled proliferation (1). Nowadays, cancer is an important public health problem in all countries (2, 3). For decades, conventional chemotherapy has been the most common type of anticancer pharmacotherapy (4). Cancer chemotherapy has been one of the major advances in the field of medicine in the last few decades. However, drugs administered for chemotherapy have a narrow therapeutic index and therefore, there is a high incidence of unwanted side effects (5, 6).

1,3,4-Thiadiazole is a five-membered ring system that has gained prominence by exhibiting a wide variety of biological activities. It has interesting pharmacophores that display a broad spectrum biological activity. The lower toxicity and *in vivo* stability of 1,3,4-thiadiazole nucleus are attributed to its aromaticity. 1,3,4-Thiadiazole has exhibited potential antiglaucoma, antiinflammatory, antitumor, antiulcer, antibacterial, antiviral, analgesic, antiepileptic, antifungal and radioprotective activities. Some marketed drugs like acetazolamide (diuretic), sulfaethidole (antibacterial), cefazolin (antibacterial), etc. have 1,3,4-thiadiazole ring (7-9).

**Figure 1:**
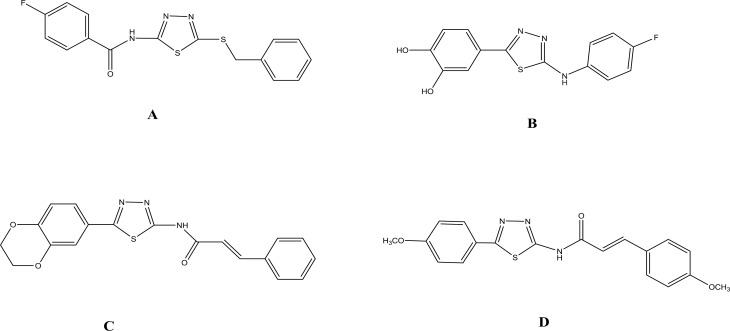
Structures of some of the 1, 3, 4-thiadiazole compounds with anticancer activity. A) *N*-(5-(Benzylthio)-1, 3, 4-thiadiazol-2-yl)-4-fluorobenzamide (10, 12). B) 4-(5-(4-Fluorophenylamino)-1,3,4-thiadiazol-2-yl) benzene-1, 2-diol (9). C) *N*-(5-(2,3-Dihydrobenzo[b][1,4]dioxin-6-yl)-1, 3,4-thiadiazol-2-yl) cinnamamide (13). D) (*E*)-3-(4-Methoxyphenyl)-*N*-(5-(4-methoxyphenyl)-1, 3, 4-thiadiazol-2-yl) acrylamide (8)

Recently, several pharmacophores containing 1,3,4-thiadiazole ring have been reported with potential anticancer activity ( Figure 1) (10-17). Radi *et al *reported the effectiveness of a new series of 1,3,4- thiadiazole derivatives as dual inhibitors of Abl and Src kinase with potential anticancer activity (12). Besides, they compared their *in vitro* potency with imatinib as Abl tyrosine kinase inhibitor. In fact, the 1,3,4-thiadiazole derivatives prepared by Radi *et al* had a binding site in the active site of Abl tyrosine kinase, similar to imatinib. In the other words, regardless of the structure of imatinib and synthesized derivatives, the structure of these 1, 3, 4-thiadiazole derivatives mimic the pharmacophoric portion  of imatinib in the receptive site (12, 14). In the present study, we also focused on the design and synthesis of new 1,3,4-thiadiazole based compounds and evaluated their *in vitro* anticancer activity against three cancer cell lines using MTT assay. 

## Materials and Methods

***Chemistry***

All chemicals including starter materials, reagents and solvents were bought from Merck and Sigma-Aldrich companies. Thin layer chromatography was done using TLC sheets from Merck Company. NMR spectra were obtained by Brucker 200  MHz and chemical shifts are expressed as δ (ppm) with tetramethylsilane (TMS) as internal standard. The IR spectra were obtained by Shimadzu 470 spectrophotometer  (potassium bromide disks). Melting points were determined using electrothermal melting point analyzer apparatus and are uncorrected. The mass spectra were run on a Finigan TSQ-70 spectrometer (Finigan, USA) at 70 eV. All cancerous cell lines were purchased from Pasteur Institute, Tehran, Iran.

**Synthesis of** ***N*** **-(5-Mercapto-1, 3, 4-thiadiazol-2-yl)-2-(4-methoxyphenyl) acetamide (2)**

In a flat bottom flask, equimolar quantities of 4-methoxyphenylacetic acid (**1** ), EDC and HOBt were mixed and stirred in acetonitrile solvent for 30 min. Then, 5-amino-1,3,4-thiadiazole-2-thiol (**2** ) was added to the mixture and stirring was continued for 24 hr. Acetonitrile was removed under reduced pressure, ethylacetate/water was added and aqueous layer was removed. Organic phase was washed three times by sodium bicarbonate, diluted sulfuric acid and brine. Drying was done by anhydrous sodium sulfate and filtration was applied for the removal of sodium sulfate. Organic layer was evaporated using rotary evaporator apparatus and a yellowish powder was obtained for compound **3**. Diethyl ether was used for washing the obtained powder (18).

mp : 135-142^0^C, Yield: 84%, ^1^H-NMR (DMSO-d_6_, 200 MHz) δ: 3.35 (s, 2H, -CH_2_CO-), 3.38 (s, 1H, -SH), 3.79 (s, 3H, -OCH_3_), 7.76 (d, 2H, *J*= 8 Hz, Phenyl), 8.02 (d, 2H, *J*= 8 Hz, Phenyl), 13.1 (brs, NH). IR (KBr, cm^-1^): 3421, 3147, 2924, 2854, 1697, 1570, 1512, 1303, 1249, 1064, 794. MS (*m/z*): M^+^: 281 (45), 238 (25), 148 (90), 121 (100), 91 (25), 78 (30).

***General procedure of the synthesis of compounds*** **3a-3l**

**Scheme 1:**
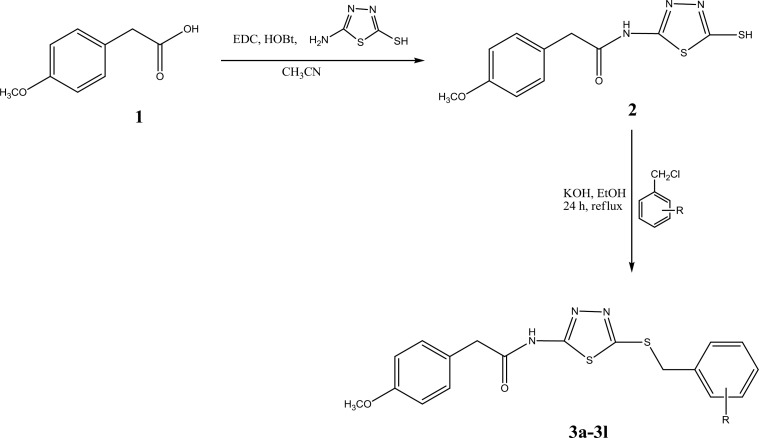
Synthesis of compounds **3a-3l**

All intended **3a-3l** derivatives were synthesized according to scheme 1. Equimolar quantities of compound **3** and potassium hydroxide were mixed and heated for 5 min and then appropriate benzyl chloride derivative was added. Reflux condition was applied for 24 hr and thin layer chromatography (TLC) was used for determining the reaction end. The reaction medium was cooled by crushed ice and cool water. A white precipitate was obtained for all compounds **3a-3l**.

***N*** **-(5-(2-Chlorobenzylthio)-1,3,4-thiadiazol-2-yl)-2-(4-methoxyphenyl) acetamide (3a)**

mp: 190-192^°^C, Yield: 55%, ^1^H-NMR (DMSO-d_6_, 200 MHz) δ: 3.38 (s, 2H, -CH_2_CO-), 3.37 (s, 3H, - OCH_3_), 4.58 (s, 2H, S-CH_2_-), 6.93 (d, 2H, *J*= 8 Hz, 4-Methoxyphenyl), 7.27 (d, 2H, *J*= 8 Hz, 4-Methoxyphenyl), 7.37 (m, 2H, 2-Chlorophenyl), 7.48 (m, 2H, 2-Chlorophenyl), 12.89 (s, NH). IR (KBr, cm^-1^): 3440, 3155, 2921, 1690, 1567, 1509, 1321, 1247, 1134, 1031, 825, 760.

***N*** **-(5-(3-Chlorobenzylthio)-1,3,4-thiadiazol-2-yl)-2-(4-methoxyphenyl) acetamide (3b)**

mp: 180-182^°^C, Yield: 56%, ^1^H-NMR (DMSO-d_6_, 200 MHz) δ: 3.30 (s, 2H, -CH_2_CO-), 3.69 (s, 3H, -OCH_3_), 4.50 (s, 2H, -S-CH_2_-), 6.85 (d, 2H, *J*= 8 Hz, 4-Methoxyphenyl), 7.19 (d, 2H, *J*= 8 Hz, 4-Methoxyphenyl), 7.28 (m, 2H, 2-Chlorophenyl), 7.45 (m, 2H, 2-Chlorophenyl), 12.81 (brs, NH). IR (KBr, cm^-1^): 3156, 2922, 1691, 1604, 1567, 1509, 1470, 1444, 1321, 1247, 1133, 1033, 826, 760.

***N*** **-(5-(4-Chlorobenzylthio)-1,3,4-thiadiazol-2-yl)-2-(4-methoxyphenyl) acetamide (3c)**

mp: 210^°^C, Yield: 56%, ^1^H-NMR (DMSO-d_6_, 200 MHz) δ: 3.38 (s, 2H, -CH_2_CO-), 3.76 (s, 3H, -OCH_3_), 4.50 (s, 2H, -S-CH_2_-), 6.92 (d, 2H, *J*= 8 Hz, 4-Methoxyphenyl), 7.26 (d, 2H, *J*= 8 Hz, 4-Methoxyphenyl), 7.39 (s, 4H, 4-Chlorophenyl), 12.86 (brs, NH). IR (KBr, cm^-1^) ύ: 3375, 3125, 2924, 2850, 1693, 1512, 1458, 1296, 1253, 1159, 1051, 825. MS (*m/z*, %): M^+^: 405 (10), M^+^+1: 406 (12), M^+^+2: 407 (10), 148 (90), 121 (100), 91 (20), 83 (20), 69 (20), 57 (25). 

**2-(4-Methoxyphenyl)-** ***N*** **-(5-(2-nitrobenzylthio)-1,3,4-thiadiazol-2-yl) acetamide (3d)**

mp: 159-162^°^C, Yield: 63%, ^1^H-NMR (DMSO-d_6_, 200 MHz) δ: 3.38 (s, 2H, -CH_2_CO-), 3.76 (s, 3H, -OCH_3_), 4.78 (s, 2H, -CH_2_CO-), 6.92 (d, 2H, *J*= 8 Hz, 4-Methoxyphenyl), 7.26 (d, 2H, *J*= 8 Hz, 4-Methoxyphenyl), 7.69 (m, 3H, 2-Nitrophenyl), 8.09 (m, 1H, 2-Nitrophenyl), 12.88 (brs, NH). IR (KBr, cm^-1^): 3430, 2923, 1685, 1610, 1558, 1523, 1341, 1306, 1036, 788.

**2-(4-Methoxyphenyl)-** ***N*** **-(5-(3-nitrobenzylthio)-1,3,4-thiadiazol-2-yl) acetamide (3e)**

mp: 213^°^C, Yield: 59%, ^1^H-NMR (DMSO-d_6_, 200 MHz) δ: 3.35 (s, 2H, -CH_2_CO-), 3.65 (s, 3H, -OCH_3_), 4.63 (s, 2H, -S-CH_2_-), 6.88 (d, 2H, *J*= 8 Hz, 4-Methoxyphenyl), 7.22 (d, 2H, *J*= 8 Hz, 4-Methoxyphenyl), 7.79 (t, 1H, C-H_5_, 3-Nitrophenyl), 7.86 (d, 1H, C-H_6_, 3-Nitrophenyl), 8.14 (d, 1H, C-H_4_, 3-Nitrophenyl), 8.30 (s, 1H, C-H_2_, 3-Nitrophenyl), 12.84 (s, NH). IR (KBr, cm^-1^): 3443, 2901, 1694, 1553, 1519, 1439, 1351, 1247, 1170, 1089, 828, 715.

**2-(4-Methoxyphenyl)-** ***N*** **-(5-(4-nitrobenzylthio)-1, 3, 4-thiadiazol-2-yl) acetamide (3f)**

mp: 168-174^°^C, Yield: 72%, ^1^H-NMR (DMSO-d_6_, 200 MHz) δ: 3.39 (s, 2H, -CH_2_CO-), 3.76 (s, 3H, -OCH_3_), 4.65(s, 2H, -S-CH_2_-), 6.92 (d, 2H, *J*= 8 Hz, 4-Methoxyphenyl), 7.26 (d, 2H, *J*= 8 Hz, 4-Methoxyphenyl), 7.70 (d, 2H, *J*= 8 Hz, 4-Nitrophenyl), 8.22 (d, 2H, *J*= 8 Hz, 4-Nitrophenyl), 12.87 (brs, NH). IR (KBr, cm^-1^) ύ: 3260, 3150, 3050, 2926, 2854, 1689, 1568, 1519, 1348, 1301, 1257, 1150, 848. MS (*m/z*, %): M^+^+1: 417 (10), M^+^: 416 (12), 148 (100), 121 (90).

***N*** **-(5-(3-Methoxybenzylthio)-1,3,4-thiadiazol-2-yl)-2-(4-methoxyphenyl) acetamide (3g)**

mp: 150-154°C, Yield: 59%, ^1^H-NMR (DMSO-d_6_, 200 MHz) δ: 3.39 (s, 2H, -CH_2_CO-), 3.76 (s, 6H, -OCH_3_, 4-Methoxyphenyl, 3-Methoxyphenyl), 4.48 (s, 2H, -S-CH_2_-), 6.88 (d, 2H, *J*= 8 Hz, 4-Methoxyphenyl), 6.95-7.01 (m, 5H, 3-Methoxyphenyl), 7.26 (d, 3H, *J*= 8 Hz, 4-Methoxyphenyl), 12.86 (brs, NH). IR (KBr, cm^-1^): 3432, 3155, 2904, 2839, 1703, 1612, 1562, 1514, 1435, 1301, 1249, 1170, 1087, 1047, 829.

**Table 1:** Cytotoxic effects, IC_50_ (µM) of compounds **3a-3l** toward three cancer cell lines

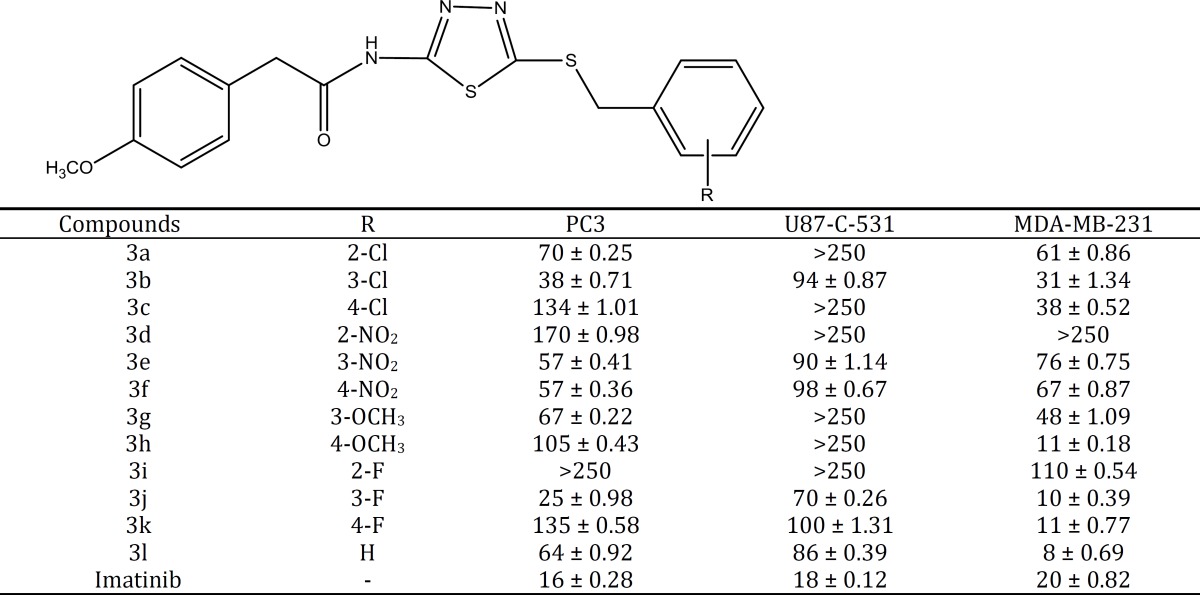

***N*** **-(5-(4-Methoxybenzylthio)-1,3,4-thiadiazol-2-yl)-2-(4-methoxyphenyl) acetamide (3h)**

mp: 165-170°C, Yield: 57%, ^1^H-NMR (DMSO-d_6_, 200 MHz) δ: 3.3 (s, 2H, -CH_2_CO-), 3.72 (s, 3H, -OCH_3, _4-Methoxyphenyl), 3.77 (s, 3H, -OCH_3_, 4-Methoxyphenyl), 4.46 (s, 2H, -S-CH_2_-), 6.92 (d, 2H, *J*= 8 Hz, 4-Methoxyphenyl), 7.23-7.37 (m, 6H, aromatic), 12.85 (brs, NH). MS (m/z): 401, 368, 281, 193, 148, 121, 78.

***N*** **-(5-(2-Fluorobenzylthio)-1,3,4-thiadiazol-2-yl)-2-(4-methoxyphenyl) acetamide (3i)**

mp: 155-159°C, Yield: 54%, ^1^H-NMR (DMSO-d_6_, 200 MHz) δ: 3.38 (s, 2H, -CH_2_CO-), 3.76 (s, 3H, -OCH_3_), 4.51 (s, 2H, -S-CH_2_-), 6.92 (d, 2H, *J*= 8 Hz, 4-Methoxyphenyl), 7.30 (d, 2H, *J*= 8 Hz, 4-Methoxyphenyl), 7.28-7.52 (m, 4H, 2-Fluorophenyl), 12.88 (s, NH). IR (KBr, cm^-1^): 3430, 2922, 2853, 1692, 1564, 1510, 1248, 1136, 1031, 759. 

***N*** **-(5-(3-Fluorobenzylthio)-1,3,4-thiadiazol-2-yl)-2-(4-methoxyphenyl) acetamide (3j)**

mp: 155-158°C, Yield: 56%, ^1^H-NMR (DMSO-d_6_, 200 MHz) δ: 3.30 (s, 2H, -CH_2_CO-), 3.68 (s, 3H, -OCH_3_), 4.44 (s, 2H, -S-CH_2_-), 6.84 (d, 2H, *J*= 8 Hz, 4-Methoxyphenyl), 7.18 (d, 2H, *J*= 8 Hz, 4-Methoxyphenyl), 7.07-7.34 (m, 4H, 3-Fluorophenyl), 12.78 (brs, NH). IR (KBr, cm^-1^): 3158, 2842, 1686, 1565, 1514, 1405, 1255, 1170, 827, 788.

***N*** **-(5-(4-Fluorobenzylthio)-1,3,4-thiadiazol-2-yl)-2-(4-methoxyphenyl) acetamide (3k)**

mp: 184-186°C, Yield: 61%, ^1^H-NMR (DMSO-d_6_, 200 MHz) δ: 3.38 (s, 2H, -CH_2_CO-), 3.76 (s, 3H, -OCH_3_), 4.50 (s, 2H, -S-CH_2_-), 6.92 (d, 2H, *J*= 8 Hz, 4-Methoxyphenyl), 7.26 (d, 2H, *J*= 8 Hz, 4-Methoxyphenyl), 7.24 (t, 2H, 4-Fluorophenyl), 7.47 (t, 2H, 4-Fluorophenyl), 12.86 (brs, NH). IR (KBr, cm^-1^) ύ: 3395, 3160, 2937, 2854, 1985, 1562, 1510, 1361, 1290, 1246, 1168, 1026, 817. MS (*m/z*, %): M^+^: 389 (30), 150 (40), 149 (100), 148 (90), 122 (80), 121 (90), 109 (40), 91 (20), 71 (60), 55 (30).

***N*** **-(5-(Benzylthio)-1,3,4-thiadiazol-2-yl)-2-(4-methoxyphenyl) acetamide (3l)**

mp: 177-180°C, Yield: 72%, ^1^H-NMR (DMSO-d_6_, 200 MHz) δ: 3.29 (s, 2H, -CH_2_CO-), 3.68 (s, 3H, -OCH_3_), 4.42 (s, 2H, -S-CH_2_-), 6.84 (d, 2H, *J*= 8 Hz, 4-Methoxyphenyl), 7.26 (d, 2H, *J*= 8 Hz, 4-Methoxyphenyl), 7.16-7.36 (m, 5H, aromatic). IR (KBr, cm^-1^) ύ: 3350, 3150, 3030, 2945, 2887, 1716, 1666, 1556, 1396, 1325, 1244, 1165, 1124, 1080, 1022, 790, 696. MS (*m/z*, %): M^+^+1: 372 (58), M^+^: 371 (60), 225 (20), 148 (100), 121 (90), 91 (40).

***MTT assay***

The derivatives of 1,3,4-thiadiazole (compounds **3a-3l**) were assessed regarding their cytotoxic activity at 0.1-250 µg/ml in three human cancer cell lines; PC3 cell (prostate cancer), U87-C-531 (gliobalstoma) and MDA-MB-231 (breast cancer). Cells from different cell lines were seeded in 96-well plates at the density of 10×10^4^viable cells per well and incubated for 24 hr to allow cell attachment. Cells were then incubated for another 24 hr (depending on the cell cycle of each cell line) with various concentrations of compounds **3a-3l**. Cells were then washed in PBS, and 20 μl of MTT (3- (4,5-dimethylthiazol-2-yl)-2,5-diphenyl tetrazolium bromide) solution (5 mg/ml) was added to each well. An additional 4 hr incubation at 37°C was done, and then the medium was discarded. Dimethyl sulfoxide (60 μl) was added to each well, and the solution was vigorously mixed to dissolve the purple tetrazolium crystals. The absorbance of each well was measured by ELISA plate reader (Anthous 2020, Austria) at the test wavelength of 550 nm against the standard reference solution at 690 nm. The amount of purple formazan production is proportional to the number of viable cells (18).

## Results

Compound **3** was used for preparing compounds **3a-3l**, as an intermediate material. Compound **3** and compounds **3a-3l** were synthesized at room temperature and under reflux condition, respectively. Compound 3 was prepared with high yield (84%) as a yellowish powder and an average yield (54-72%) was obtained for final derivatives in the form of a creamy to white powder. Melting point of intermediate and final compounds was reported. A range of 154-213C was recorded for final compounds. ^1^H-NMR, IR and MS spectra for the intermediate and the final compounds were obtained. 

The cytotoxicity of all synthesized compounds against three cancerous cell lines was evaluated by MTT assay (Table 1). Overall, all compounds **3a-3l** afforded higher cytotoxic activity against MDA-MB-231, breast cancer cell line and lower cytotoxic activity against U87-C-531, glioblastoma cell line. Substitution of chlorine atom at position 3 (*meta*) of the phenyl ring resulted in a higher cytotoxic property than other positions of the phenyl ring. Compounds **3a-3c** with chlorine moiety rendered better activity against MDA-MB-231 cell line in comparison with other cell lines. Replacement of chlorine moiety with nitro group decreased the cytotoxic effect of compounds **3d-3f** against all three cell lines. Nitro group substituent can be more beneficial at positions 3 (*meta*) and 4 (para) of the phenyl ring compared to position 2 (*ortho*). Substitution of methoxy at position 4 in compound **3h** (IC_50_= 11 ± 0.18 µM) led to a cytotoxic effect against MDA-MB-231 cell line compared to imatinib (IC_50_= 20 ± 0.82 µM). As with chlorine, a similar trend like was also observed for fluorine substituent. Position 3 (*meta*) of the phenyl ring was the best position for fluorine to render its cytotoxic activity in compounds **3i-3k**. Compounds **3i-3k** like other synthesized compounds in this series exhibited higher cytotoxic activity against MDA-MB-231 breast cancer cell line compared to cytotoxic effect against PC3 and U87-C-531 cell lines. Compound **3j **(IC_50_= 10 ±0.39 µM) with *ortho* fluorine moiety and compound 3k (IC_50_= 11 ± 0.77 µM) with *para* fluorine moiety demonstrated higher cytotoxic activity against MDA-MB-231 cell line as compared with imatinib (IC_50_= 20 µM). Phenyl ring without any substituent (compound 3l) had the most beneficial cytotoxic effect (IC_50_= 8 ± 0.69 µM) in all synthesized compounds of this series. 

## Discussion

A new series of 1,3,4-thiadiazole based compounds was synthesized and their anticancer property was assessed by MTT assay, *in vitro*. Against PC3 cell line, Compound **3j** with *meta* fluorine moiety showed the best cytotoxic effects. On the other hand, compound **3i **with *ortho* fluorine substituent exerted the lowest anticancer activity in this series. This trend was also observed against U87-C-531 and MDA-MB-231 cell lines. U87-C-531 cell line was the most resistant cell line to the tested compounds in MTT assay and the recorded IC_50_ were not significant. 

Overall, replacing electron withdrawing groups at position *ortho* of the phenyl ring was not capable of enhancing the cytotoxic activity. Fluorine moiety as an electron withdrawing group demonstrated a high cytotoxic potency when substituted at positions *meta* and *para*. Interestingly, compound **3l** without any electron withdrawing and donating moiety on the phenyl ring, rendered the highest anticancer potency toward MDA-MB-231, PC3 and U87-C-531 cell lines (IC_50_= 8 ± 0.69 µM). Methoxy moiety as applied in compound **3h** as an electron donating group caused an increase in the cytotoxic potency against MDA-MB-231 cell line when it was located at *para* position of the phenyl ring. 

According to the obtained data from MTT assay, all compounds (**3a-3l**) were more toxic toward MDA-MB-231 breast cancer cell line in comparison with other cell lines. It is also notable that four synthesized compounds 3 hr (IC_50_= 11 ± 0.18 µM), **3j **(IC_50_= 10 ± 0.39 µM), **3k **(IC_50_= 11 ± 0.77 µM) and **3l **(IC_50_= 8 ± 0.69 µM) exhibited higher cytotoxic activity against MDA-MB-231 cell line compared to imatinib (IC_50_= 20 ± 0.69 µM) as the reference drug.

## Conclusion

According to the obtained results, synthesized compounds could be proposed as potential anticancer lead compounds. The presented compounds exhibited robust anticancer activity against MDA-MB-231 (breast cancer) cell line,* in vitro*. Therefore, further investigation about the probable mechanism of these derivatives and also their *in vivo *activity evaluation is needed to be done in future studies. According to our data, *ortho* positioning of moieties was detrimental for cytotoxic activity. It suggests focusing on *meta* and *para* derivatives in the next explorations. Regarding the PC3 and U87-C-531 cell lines, more structural modification is necessary to enhance the cytotoxic potency. Overall, synthesis of new 1,3,4-thiadiazole derivatives can lead to the production of compounds with potential anticancer property, especially against breast cancer.
